# Colovesical Fistula: An Unusual Complication of Cytomegalovirus Colitis

**DOI:** 10.7759/cureus.1426

**Published:** 2017-07-05

**Authors:** Talal Asif, Badar Hasan, Alisa Likhitsup, David Bamberger

**Affiliations:** 1 Department of Internal Medicine, University of Missouri Kansas City (UMKC); 2 Fellow, Department of Gastroenterology, University of Missouri Kansas City (UMKC); 3 Professor of Medicine, Section Chief Infectious Diseases, University of Missouri Kansas City (UMKC)

**Keywords:** cmv colitis, cmv enteric fistula, cmv colovesical fistula, hiv complication

## Abstract

Cytomegalovirus (CMV) is a double-stranded DNA virus that is associated with clinically significant disease in patients with advanced immunosuppression, particularly those with human immunodeficiency virus (HIV) infection and acquired immunodeficiency syndrome (AIDS). End-organ disease with CMV is classically associated with a CD4 cell count less than 50 cells/microliter. CMV colitis is the second most common manifestation of end-organ disease in this patient population. CMV-associated enteric fistula is a rare complication that has been described in only a few case reports in the literature. These cases describe gastrocolic, enterocutaneous, enterocolic, rectovaginal, and colocutaneous fistulae. However, colovesical fistula has not been described previously. Here, we report the first case of CMV-associated colovesical fistula in a patient with HIV infection and AIDS.

## Introduction

Cytomegalovirus (CMV) is a large DNA virus of the herpes family [1]. It is a very ubiquitous virus with rates of CMV seroprevalence estimated at 50% in the adult population [1]. Infection in an immunocompetent host is generally asymptomatic. However, infection in immunocompromised patients is associated with clinically significant diseases especially in patients with human immunodeficiency virus (HIV) and organ transplants.

Patients with HIV and acquired immunodeficiency syndrome (AIDS) with CD4 cell counts below 50 cells/microliter are at high risk for CMV infection involving multiple organs including the gastrointestinal tract [2]. CMV colitis is the second most common manifestation of end organ involvement after CMV retinitis [1]. CMV colitis most commonly presents as mild to severe persistent diarrhea but in severe cases, it can progress to perforating colitis, hemorrhagic proctocolitis, and toxic megacolon [3]. However, CMV-related enteric fistulas are exceedingly rare and have been described in only four case reports [1].

Here, we present a very rare case of CMV-related colovesical fistula in a patient with HIV infection and AIDS who was initially misdiagnosed as having Crohns’ disease. According to our detailed literature review, this is the first case of a CMV-related colovesical fistula in a patient with HIV infection and AIDS [1]. Through this case, we aim to raise awareness of the potential for CMV-related enteric fistulas so that they can be recognized and treated at an earlier stage. We also provide a comprehensive management approach to help guide current practicing physicians.

## Case presentation

A 40-year-old African-American female patient with a past medical history of treatment for naïve HIV infection and polysubstance abuse presented to us with the chief complaint of abdominal pain and diarrhea.

**Figure 1:**
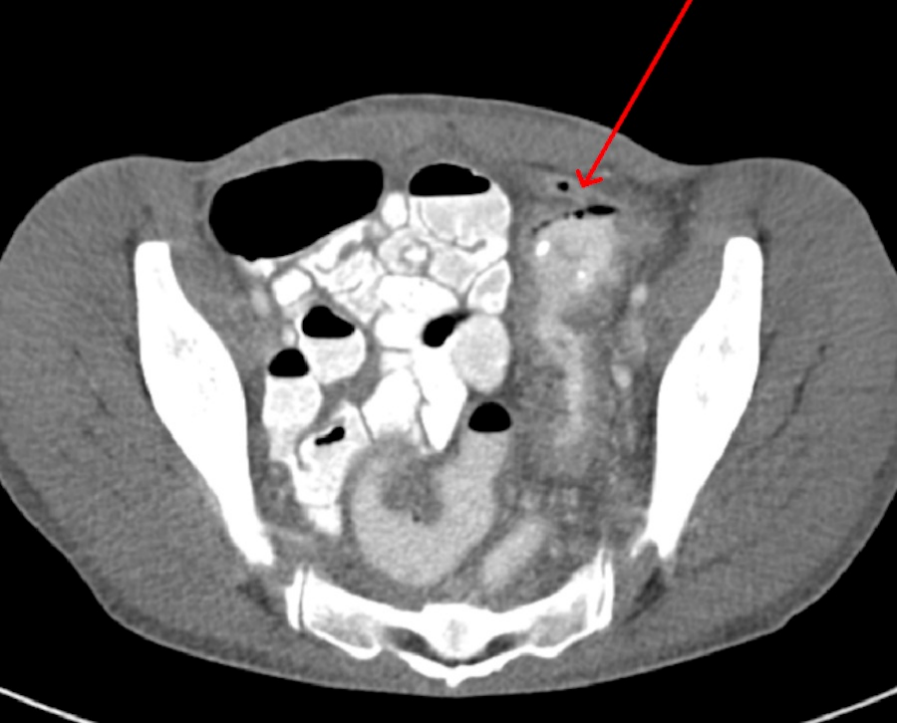
Computed tomographic (CT) scan of the abdomen and pelvis showing small tract (red arrow) extending from the sigmoid colon towards the bladder dome suggestive of fistulous communication

The patient’s history dates back to one year prior when she had presented with similar complaints. At that time, the patient complained of watery diarrhea, left lower quadrant abdominal pain, and fecaluria for one month. Her stool clostridium difficile antigen test was first obtained and it came back positive. The urine drug screen was positive for cocaine and methamphetamines. A computed tomography (CT) scan of the abdomen was done that showed a segmental colitis involving the sigmoid colon and small fistulous tract extending from the sigmoid colon towards the bladder dome (Figure 1).

The patient was started on oral vancomycin and the gastroenterology (GI) team was consulted. Owing to her cocaine use, the patient was deemed unsafe for colonoscopy and she underwent flexible sigmoidoscopy which showed multiple sigmoid colon ulcers. Numerous biopsies were performed. At this point, it was thought that the patient might have superimposed clostridium difficile infection in the background of underlying fistulous Crohn's disease. The GI team recommended continuing the patient on oral vancomycin and obtaining anti-neutrophil cytoplasmic antibody (ANCA) and anti-saccharomyces cerevisiae antibodies (ASCA) with follow-up as an outpatient in the inflammatory bowel disease (IBD) and infectious diseases clinic. The patient’s ANCA and ASCA came back negative. Sigmoid colon biopsy results showed cryptitis, crypt abscesses, and low purulent exudate consistent with active colitis. Unfortunately, the patient never returned to the clinic and was lost to follow-up.

One year later, the patient presented again with diarrhea and abdominal pain. She had similar complaints of non-radiating left lower quadrant abdominal pain and watery diarrhea with passage of feces in the urine (fecaluria). The patient reported that after discharge from the hospital on the previous hospitalization, her diarrhea had initially improved but it never resolved and for the past two months, she had experienced worsening of her symptoms.

On arrival, the patient had a blood pressure of 121/75 mmHg, heart rate of 98/minute, temperature of 98°F and respiratory rate of 18/minute. The patient had tenderness in her left lower quadrant to light palpation with no rigidity or guarding. Bowel sounds were normal. Rest of the cardiovascular, respiratory, and neurological exam was unremarkable.

**Figure 2:**
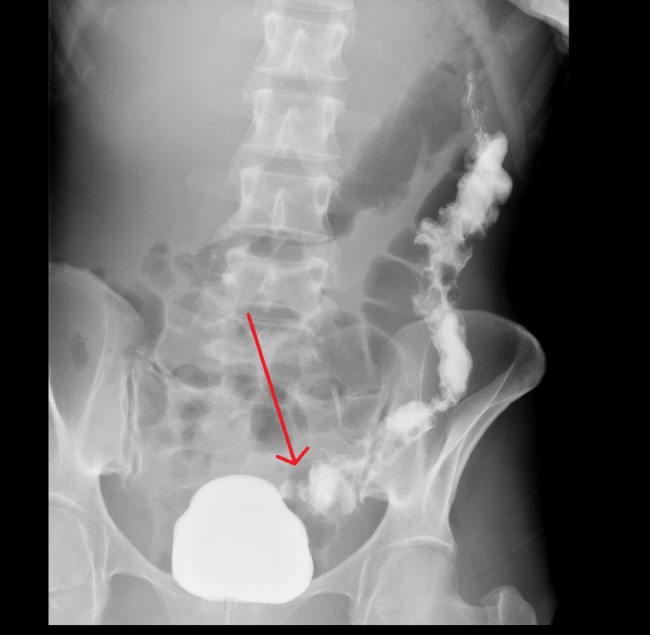
X-ray cystogram showing colovesical fistula (red arrow) connecting the bladder to the proximal sigmoid colon

Baseline labs including complete blood count, basic metabolic panel, and liver function tests were within normal limits. Repeat CD4 count was less than 20 cells/microliter and HIV viral load was 2,76,4591 copies/mL. Erythrocyte sedimentation rate (ESR) was elevated at 60 mm/hr and stool for clostridium difficile toxin was negative. The CT scan of the abdomen and pelvis with oral and intravenous contrast re-demonstrated the thickening of the left colonic bowel wall concerning for colitis. The patient underwent cystogram which showed colovesical fistula connecting to the proximal sigmoid colon (Figure 2).

The GI team recommended a colonoscopy to evaluate the etiology and extent of the colitis. On colonoscopy, the patient’s sigmoid colon appeared erythematous with friable ulceration. A moderate sized opening was also seen representing a fistula and multiple biopsies were obtained from the inflamed segment surrounding the fistulous opening.

**Figure 3:**
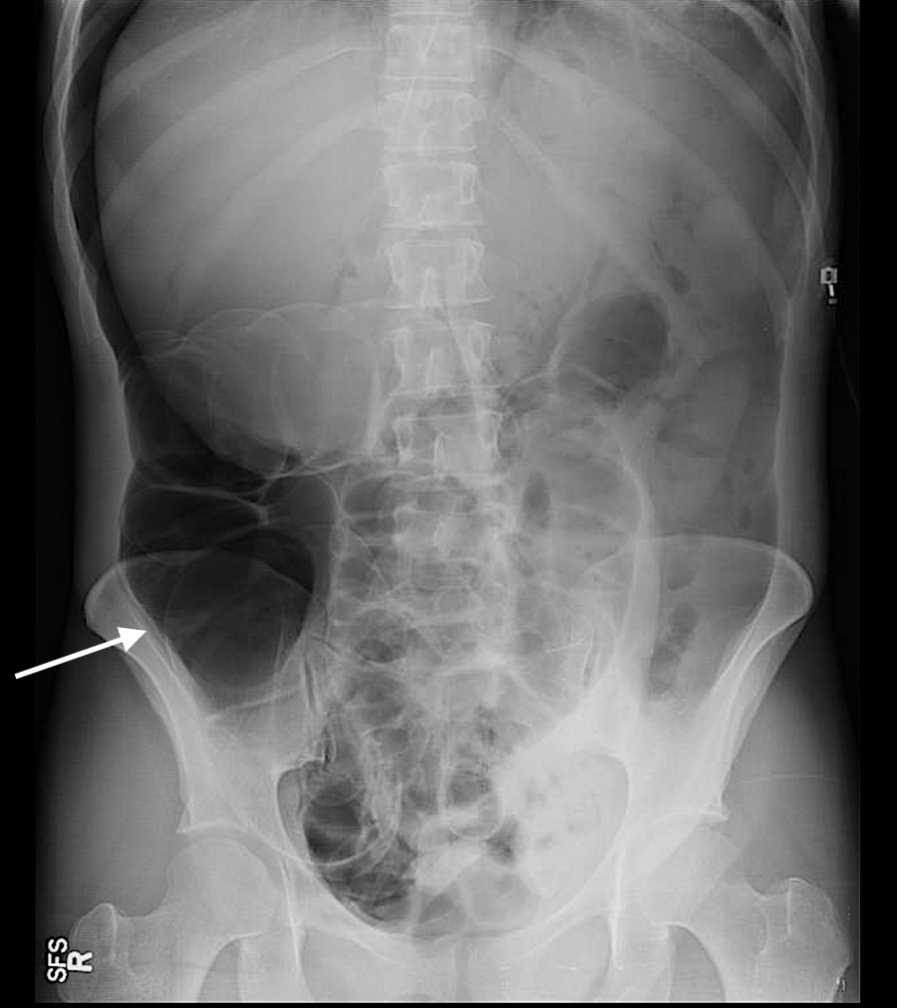
X-Ray abdomen A significant amount of free air is seen within the abdomen. Given the patient's history of recent colonoscopy and acute abdominal pain, this likely represented intestinal perforation.

Post procedure, the patient complained of severe diffuse abdominal pain. An urgent X-ray of the abdomen was obtained which showed free air under the diaphragm (Figure 3).

The patient was emergently taken to the operating room (OR) where she underwent exploratory laparotomy and was found to have a perforation in her sigmoid colon. An intraoperative urology consult was obtained for the evaluation of colovesical fistula. The patient underwent cystorrhaphy, sigmoid bowel resection with the creation of Hartmann's pouch. She was transferred to the surgical intensive care unit.

**Figure 4:**
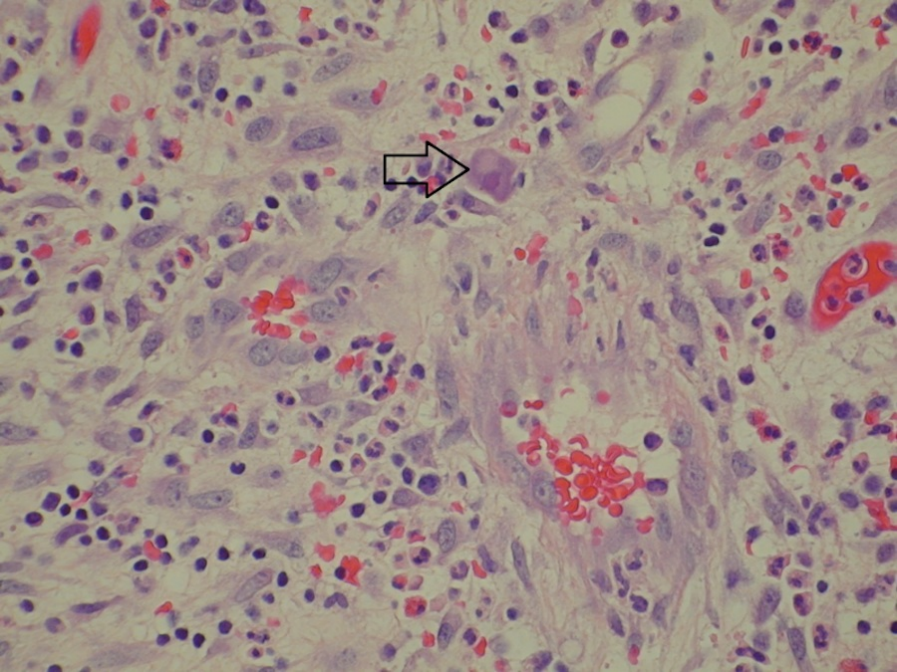
Haemotoxylin and eosin (H&E) stain: histopathology slide of the fistulous tract in the urinary bladder Fibroadipose tissue with fibrinopurulent exudate, granulation tissue, and congestion is seen on the slide. Nuclear inclusions consistent with cytomegalovirus (CMV) infection (arrow) are also seen.

Results of biopsies from the urinary bladder, especially from the fistulous tract, showed intranuclear inclusions consistent with CMV infection (Figure 4).

**Figure 5:**
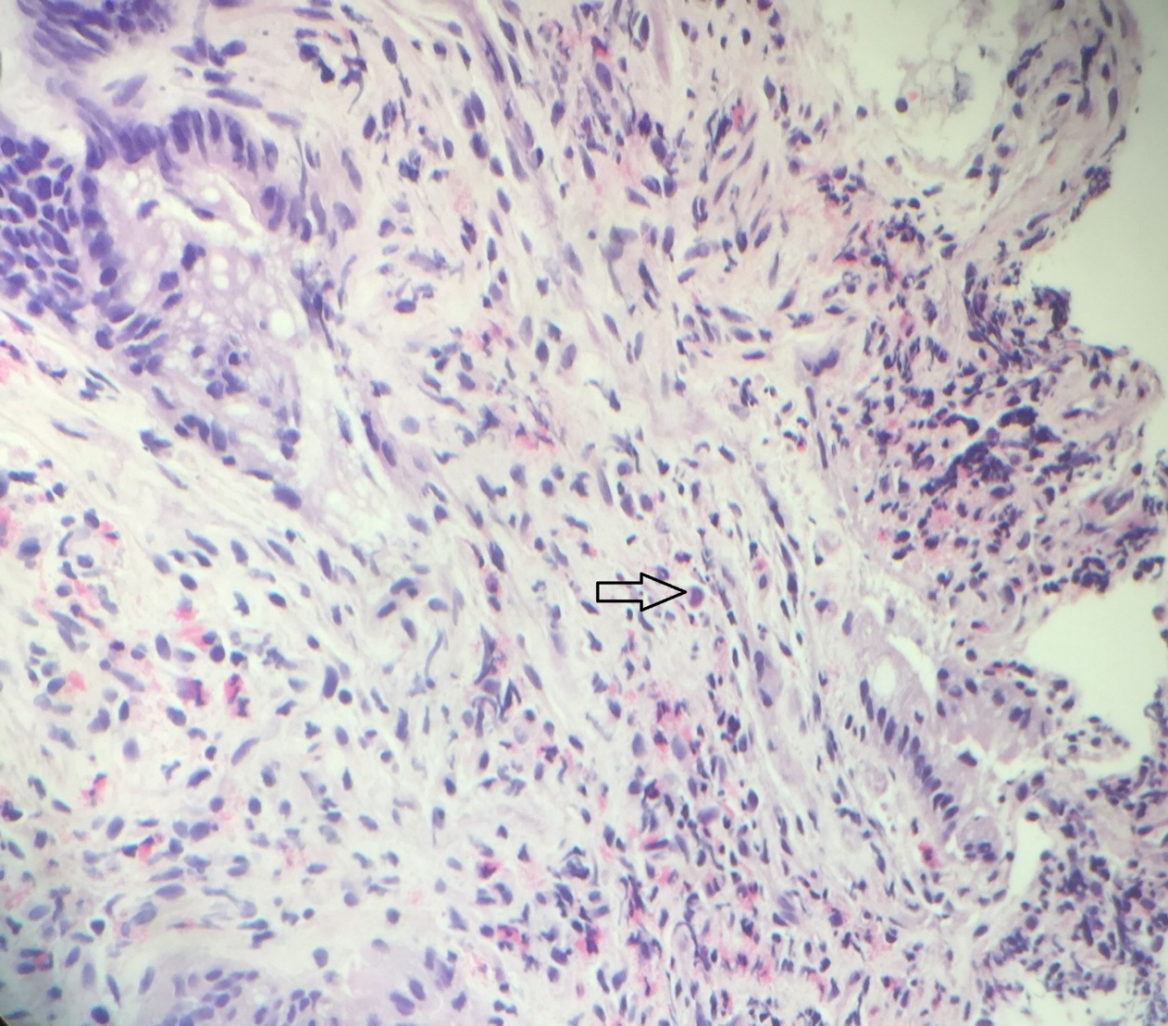
Haemotoxylin and eosin (H&E) stain: slide from colon and sigmoid resection specimen Erosion, acute inflammation, and congestion are seen. Nuclear inclusions consistent with cytomegalovirus (CMV) infection are identified (black arrow).

Histopathological analysis of slides obtained from the resected omentum, sigmoid colon, colostomy site, and sigmoid ulcer also revealed mucosal inflammation, tissue necrosis, and intranuclear inclusions characterizing CMV infection (Figure 5).

**Figure 6:**
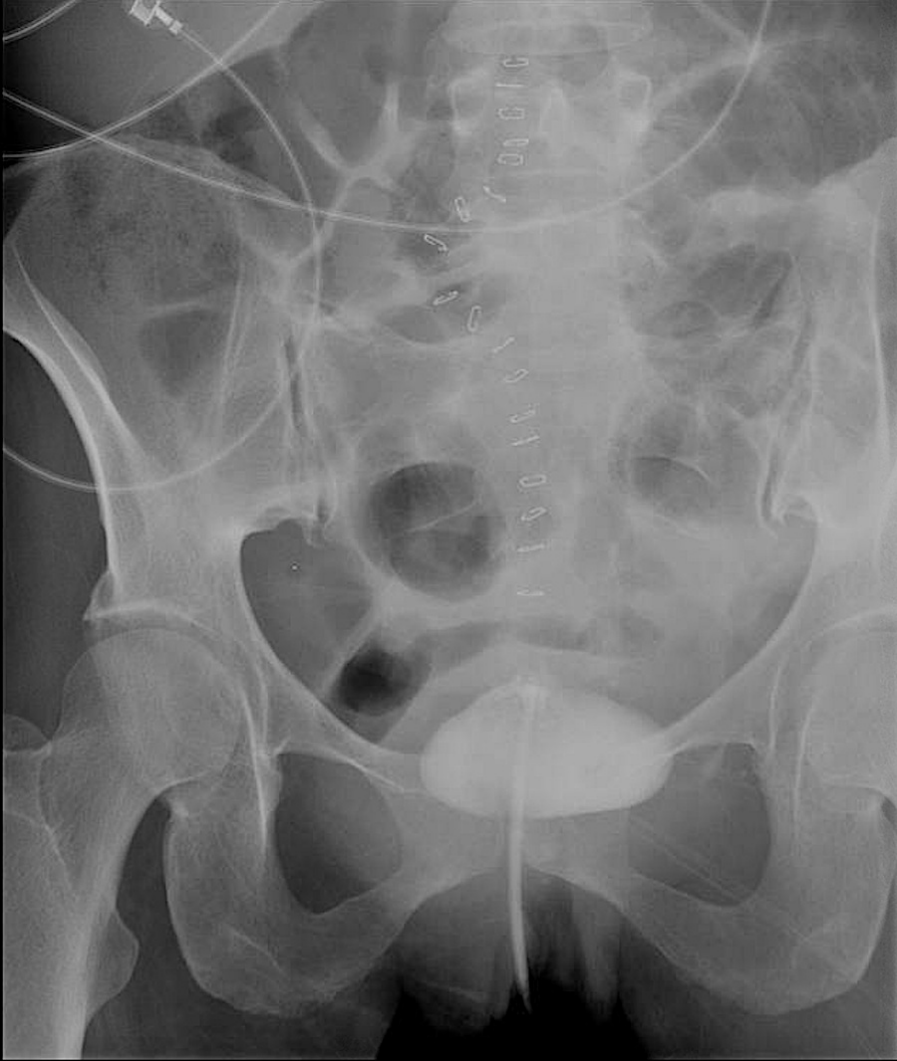
XR cystogram after surgery showing no evidence of bladder leak or colovesical fistula

There was no granuloma or colitis seen. Serum CMV DNA polymerase chain reaction (PCR) was significant for 6,261 copies/mL. The infectious disease team was consulted and it was determined that in the context of the patient's HIV infection, AIDS and resultant immunosuppression, CMV infection was contributing to chronic diarrhea, abdominal pain, and formation of a colovesical fistula. The patient was initiated on intravenous ganciclovir 5 mg/kg every twelve hours followed by valganciclovir 900 mg twice a day once she was able to tolerate oral intake. Her diarrhea improved with treatment. Prior to discharge, a repeat cystogram was obtained which did not show bladder leak or colovesical fistula (Figure 6).

The patient was discharged to a skilled nursing facility with follow-up for infectious disease and general surgery as an outpatient.

## Discussion

CMV is an important cause of gastrointestinal disease in patients with HIV infection and AIDS. CMV disease occurs most commonly as a result of reactivation of latent infection in previously seropositive patients [2]. The risk of end-organ disease with CMV increases in states of advanced immunosuppression, especially with CD4 counts less than 50 cells/microliter [4]. The esophagus, stomach, small intestine, and colon can be affected by CMV infection [3]. CMV colitis is the second most common presentation of end organ involvement after CMV retinitis [1]. Nearly 5% to 10% of patients with CMV end-organ disease have involvement of the colon [5]. The most common presenting symptoms are severe diarrhea, abdominal pain, weight loss, and anorexia [6]. Hemorrhage and perforation are life-threatening complications that require emergent surgical intervention [1].

Colovesical fistula (CVF) represents an abnormal communication between the colon and the urinary bladder [6]. Even though CVF are uncommon, they are associated with significant morbidity and have a negative impact on the patient’s quality of life [6]. The most common causes of colovesical fistula are diverticulitis, malignancy, and Crohn’s disease [7]. CVF in CMV colitis and HIV has not been described in the literature which makes our patient’s clinical course unique and distinctive [6]. The most frequent presenting symptoms in CVF are pneumaturia and fecaluria [7].

The diagnosis of CMV colitis is based upon visualization of characteristic mucosal ulcerations on colonoscopy and the demonstration of intranuclear and intracytoplasmic inclusions on histopathology [1, 5]. Biopsies need to be taken from the base of the ulcer [3, 5]. Experts also recommend taking biopsies at the site of the fistula [1, 3]. Diagnosis of CMV-associated fistula can also be made after histopathological examination of the resected tissue [8]. Blood tests to detect CMV viremia by antigen detection, polymerase chain reaction and culture are not recommended due to their poor positive predictive value for the diagnosis of CMV end-organ disease [4].

A CT scan with oral but not intravenous contrast is the imaging modality of choice for the diagnosis of CVF [6]. Cystoscopy can be performed if associated bladder malignancy is suspected. Other investigation modalities that can be used include magnetic resonance imaging (MRI), cystogram, and barium enema [6]. Urinalysis is invariably abnormal in patients with CVF.

All patients with symptomatic CMV gastrointestinal disease should be treated with anti-CMV therapy [9]. Ganciclovir, valganciclovir, and foscarnet are the antiviral agents available for treatment [9]. Ganciclovir is the preferred agent because of the abundance of data on its efficacy and safety profile [10]. Intravenous (IV) ganciclovir is the treatment of choice followed by oral valganciclovir once the patient is able to tolerate oral medications [10]. Patients should be treated with induction therapy for 21 to 42 days or until resolution of symptoms [10].

The majority of patients who have a CMV-related gastrointestinal disease are antiretroviral therapy (ART) naïve. ART should only be initiated once CMV retinitis has been ruled out due to the risk of immune reconstitution related uveitis [10]. Patients who have concurrent retinitis should be started on ART two weeks after anti-CMV therapy has been instituted [10]. ART should be continued in patients who were previously on treatment.

The treatment of choice for CVF is predominantly surgical [6]. Due to the paucity of data in the literature about the management of CMV-related CVF, approach to surgical management has to be extrapolated from recommendations on CVF in general. Since lower CD4 counts are associated with higher perioperative mortality, the decision on the timing of surgical intervention also needs to be individualized. The extent of resection is determined by the location of this fistula and the patient’s condition [7]. Surgical options include colonic resection with primary anastomosis or colonic resection with end colostomy followed by colostomy reversal at a later stage [6].

The present case is the first report of a CVF in a patient with CMV colitis and AIDS. There are no standard guidelines for treatment of patients with this condition. The management of CVF can be challenging especially in this patient population. CVFs not only cause significant morbidity but also predispose patients with severe immunocompromise to the risk of urosepsis and death, raising the need for timely and successful therapeutic strategies. Our case not only highlights the importance of appreciation of the association of CMV colitis and CVF but also provides a successful treatment approach and recommendation to the practicing physicians. Given the large disease burden of HIV, case reports like these carry tremendous significance in setting a precedent for current practicing physicians to guide them when encountered with a challenging case like this.

## Conclusions

It is important to recognize the possibility of CMV infection in patients with HIV infection presenting with colovesical fistula. This mindfulness will enable timely institution of antiviral therapy to improve patient outcomes.
